# COVID-19 Contact Tracing and Data Protection Can Go Together

**DOI:** 10.2196/19359

**Published:** 2020-04-20

**Authors:** Johannes Abeler, Matthias Bäcker, Ulf Buermeyer, Hannah Zillessen

**Affiliations:** 1 Department of Economics University of Oxford Oxford United Kingdom; 2 Rechts- und Wirtschaftswissenschaften Johannes Gutenberg-Universität Mainz Mainz Germany; 3 Gesellschaft für Freiheitsrechte (German Society for Civil Rights) Berlin Germany

**Keywords:** COVID-19, app, contact tracing, proximity tracing, privacy, data protection, Bluetooth

## Abstract

We discuss the implementation of app-based contact tracing to control the coronavirus disease (COVID-19) pandemic and discuss its data protection and user acceptability aspects.

## Why Is Contact Tracing Useful?

The coronavirus disease (COVID-19) pandemic is the greatest public health threat that the world has seen in the last 100 years. In response, countries have introduced various levels of “lockdown” to reduce the number of new infections. Lockdowns, however, come at a great cost to workers, firms, and families. Recent epidemiological models also predict that the epidemic will start anew, once the lockdown is lifted [1].

Scientists have thus discussed a second approach to keeping the epidemic in check: app-based contact tracing. Several apps are currently in development (eg, in the United Kingdom [2], by a pan-European initiative [3], and in a joint Google and Apple venture [4]), or have already been launched (eg, in Singapore [5]).

Why would such an app be useful at all? We still don’t know many things about COVID-19. The data so far suggest, however, that about half of all infections occur before the dreaded symptoms of fever or a persistent cough appear. It is therefore not enough to quarantine people only after they show symptoms. To reduce infections, one would need to act quickly when a person is diagnosed with COVID-19 to find all people this person was in close proximity with. The risk of infection is highest if one has been within 1.5-2 m of an infected person for at least 10-15 minutes. If it could be determined who had been in such close proximity, then one could ask freshly infected, presymptomatic people to self-isolate and thus stop them from infecting more people. Mathematical models of the pandemic [6] show that fast contact tracing combined with a large-scale virus-testing program might be able to not just delay the epidemic but to stop it entirely. This would also mean that the lockdown measures currently in place around the world could be slowly loosened up again. However, such fast contact tracing is not possible manually. Only a digital, largely automatic solution would help.

## Epidemiology Meets Data Protection

Some might argue that the demands of the COVID-19 crisis justify even extreme countermeasures. After all, this is about saving the lives and preserving the health of as many people as possible. Weakening data protection might be preferable to the far-reaching restrictions of personal freedom and to the economic costs of the current lockdown. In keeping with this, many countries have started tracking their citizens’ phones and using location data to monitor the spread of the virus as well as to enforce both lockdown and early isolation restrictions. The most prominent example of this is China [7], where entry to many public places is restricted to people who can show a green health code on their smartphones and thus demonstrate they have not been in contact with a confirmed case of COVID-19. However, countries like Israel [8] or South Korea [9] use location data as well—in the former case, to enforce quarantine rules and notify the contacts of an infected person, and in the latter to warn people before they enter “high risk” zones. However, even in the face of an existential threat, we should interfere with fundamental rights as little as possible. Among the effective approaches, we should choose the one that least compromises fundamental rights. In particular, we believe that swift and efficient contact tracing is possible without collecting extensive amounts of data in a central database.

A contact tracing system can be set up in a way that would allow for most data processing to happen locally on users’ mobile phones rather than on a central server. Only the notification of users who have been in contact with an infected person would need to be coordinated centrally. Even in this case, the necessary data could be processed in a way that would effectively preclude the central server from identifying users. The system would also not require collecting any location data.

The fundamental idea is simple: it does not matter *where* people get in contact with an infected person. Be it on the bus or at work—what matters is proximity to a contagious person. This means that particularly sensitive location data, such as GPS or radio cell data, is actually neither necessary nor useful. Instead, the only data that matters is whether two people have come into close enough contact to risk an infection. One example of this would be contact tracing based on “contact points” as suggested by Yasaka et al [10]. The smartphone app they propose would allow users to create “checkpoints” by generating a QR code that can be scanned by all other app users when joining their checkpoint. If checkpoints were created for any social interaction, be it among friends and family or in public spaces like a restaurant, then the app could use both this information and voluntary notifications from users should they be diagnosed with the virus to compute transmission graphs. These graphs in turn could let every user know if there were any possible transmission paths leading up to the checkpoints they visited and thus their risk of being infected. The app would not need any location data and in fact wouldn’t even require users to register. It would, however, require the active participation of users who would need to either create or join a checkpoint whenever they get close to someone outside their household. Thus, this approach relies on high levels of vigilance and willingness to participate among at least a majority of the population—not only initially but also as the pandemic continues.

Another example for this kind of “privacy by design” COVID-19 tracing approach is the TraceTogether app [5] from the Singaporean government. Unlike the contact point system, it only requires users to enable Bluetooth on their phone. Pan-European Privacy-Preserving Proximity Tracing (PEPP-PT) by the European consortium [3], as well as Google and Apple’s recently announced joint initiative [4], are following a very similar concept. We present a slightly modified version below.

**Figure 1:**
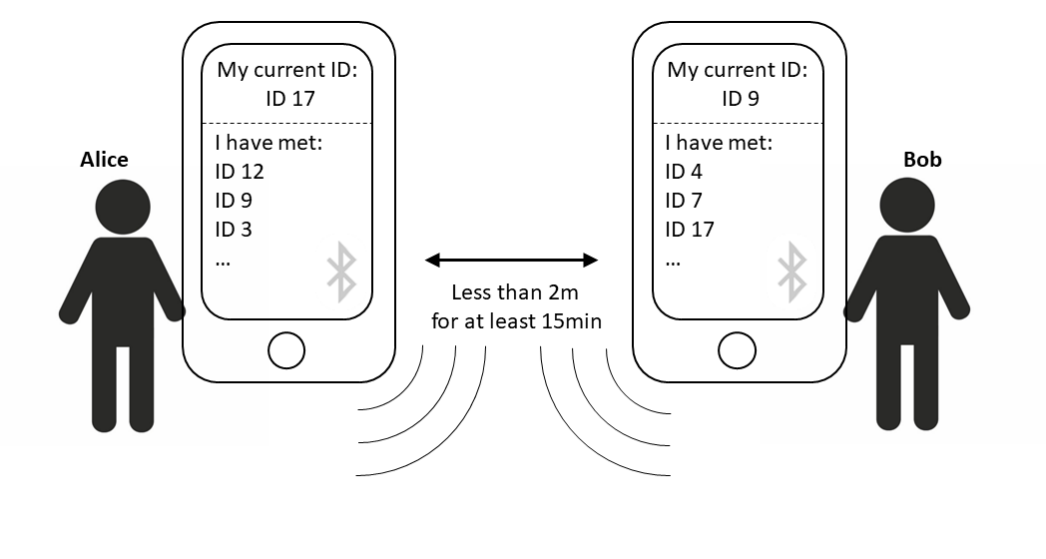
A COVID-19 tracing approach via Bluetooth. Every mobile phone stores a list of mobile phones that were within 2 m for at least 15 minutes. IDs are temporary but can be decrypted by the server.

In order to detect whether two people have come into close enough physical proximity to risk an infection, one can use Bluetooth low energy technology. The general drawback of Bluetooth—that it can only reach across a few meters—becomes an advantage here. The tracking itself would work as follows: as many people as possible voluntarily install the app on their phone. The app cryptographically generates a new temporary ID every half hour. As soon as another phone with the same app is in close proximity, both phones receive the temporary ID of the respective other app and record it. This list of logged IDs is encrypted and stored locally on the users’ phones (Figure 1).

**Figure 2:**
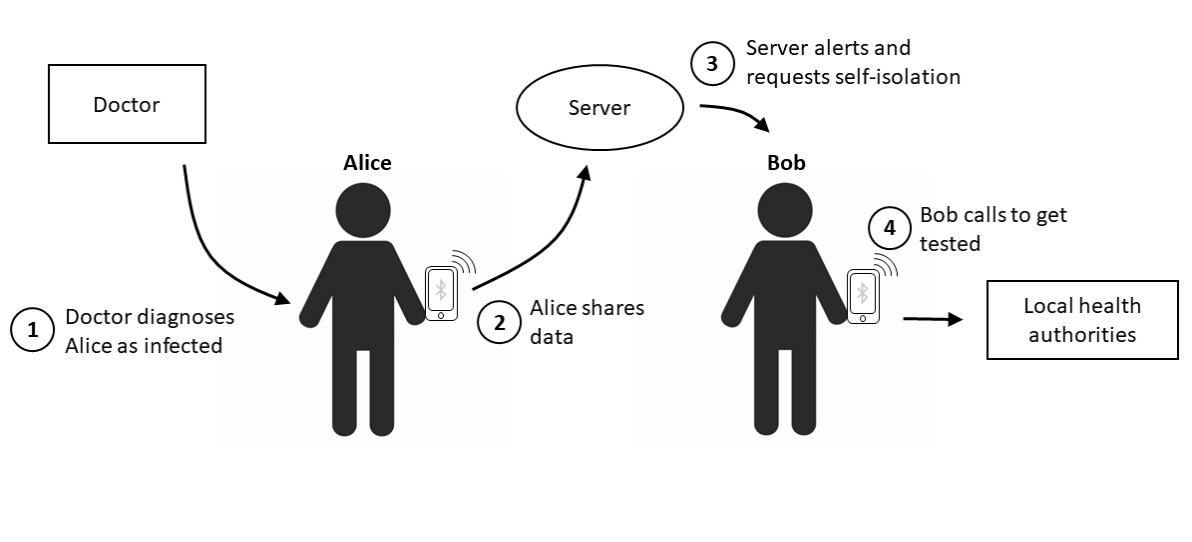
A user can share their data with the server after receiving a COVID-19 diagnosis. The server then alerts all phones that have been in close proximity to the infected phone. The alerted people would still need to contact their local health authorities, as their identity is not linked to the app.

As soon as an app user is diagnosed with COVID-19, the doctor making the diagnosis asks the user to share their locally stored data with the central server (Figure 2). If the user complies, the central server receives information on all the temporary IDs the “infected” phone has been in contact with. The server is not able to decrypt this information in a way that allows for the identification of individuals. However, it is able to notify all affected phones. This is because the server does not need any personal data to send a message to someone’s phone. The server only needs a so-called PushToken, a kind of digital address of an app installation on a particular phone. This PushToken is generated when the app is installed on the user’s phone. At the same time, the app will send a copy of the PushToken, as well as the temporary IDs it sends out over time, to a central server. The server could be hosted, for example, by the Robert Koch Institute for Germany or by the National Health Service for the United Kingdom. This way, it would be possible to contact phones solely based on temporary IDs and PushTokens whilst completely preserving the privacy of the person using the phone.

If a phone has been in close proximity to an “infected” phone, the user of that phone receives a notification together with the request to immediately go into quarantine at home. The user will then need to contact the local health authorities to get tested for the virus as soon as possible so that, depending on the outcome, the user is either able to stop quarantining or all their contacts can be informed (Figure 2).

During the entire process, no one learns the identity of the app user (eg, other users who got in close contact with them, the local health authorities, the central server) since the app is not linked to an identity. Location data is neither recorded nor stored at any point of the process.

As mentioned above, we did not come up with this concept. Singapore introduced a very similar app, and several European countries [3] are working on comparable apps as well. We do not agree with all aspects of the Singaporean app and their practice of contact tracing. For example, every app installation in Singapore is linked with the user’s telephone number, making the user identifiable—something that is not strictly necessary and thus, for data protection reasons, should be rejected. Nevertheless, we like the general concept. The recently published PEPP-PT [3] looks promising and might prove to be a legitimate implementation of the privacy-friendly tracing approach outlined above.

Such an app could implement contact tracing much more effectively than a system that relies on radio cell or location data, since neither of these two data sources permit determining a person’s position with the necessary precision of 2 m maximum. At the same time, such a concept would comply with existing data protection regulations. Finally, it would work even without users paying constant attention to potentially risky interactions as would be necessary in a contact-point system. Thus, this concept is potentially more robust to fatigue or inattentiveness.

## Data Minimization Begets Acceptance

In the case of contact tracing, the approach that requires the least amount of data also seems to be the most effective epidemiologically. This is because an app like the one described above would be better suited to determine who actually was in close proximity than any of the other proposed solutions. Moreover, even digital contact-tracing systems need users to cooperate (by installing the app and carrying their phones with them) for any chance of success. Consequently, the effectiveness of any contact-tracing system depends on public support. There is reason to believe that the level of support can be increased by opting for a data-minimizing solution. A representative survey [11] across the United States, United Kingdom, Germany, Italy, and France shows that about 70% of respondents would install an app like the one described above on their phones (disclosure: co-author JA was also the lead author of the survey study). The reason most frequently brought up against an installation is the worry that the government could use the app as an excuse for greater surveillance after the end of the epidemic. If the government wants as many people as possible to install the app, it should take these concerns seriously and refrain from using location data. Contact tracing works without it.

## Conclusion: Proportionality Instead of “Whatever It Takes”

In the current crisis, we will have to endure more and deeper encroachments on fundamental rights than we are used to. Still, there is no reason to tolerate such encroachments to a greater extent than strictly necessary. Even under the current time pressure, it is important to find solutions that minimize data processing as far as possible. We have shown above that this is possible for the case of contact tracing. As the pandemic progresses, many other challenges will emerge. For each of them, one will have to check which data processing is necessary to address them and which ones can be avoided.

Trying to find a data-minimizing solution does not just protect fundamental rights. Such solutions will often increase the effectiveness and efficiency of the respective data-processing system. Only if people trust a system—because it does not spy on them—will the system find broad support in the population.

## Abbreviations

COVID-19: coronavirus disease
PEPP-PT: Pan-European Privacy-Preserving Proximity Tracing
